# Early-Phase Drug Development in Rare Cancers: A Decade of Progress and Future Directions

**DOI:** 10.3390/cancers18203290

**Published:** 2026-10-12

**Authors:** Yin Mon Myat, Kyaw Zin Thein, Lujain Mahmood, Wen Wee Ma, Cathy Eng, Mohamed Shanshal

**Affiliations:** 1 One Brooklyn Health—Interfaith Medical Center Campus, Brooklyn, NY 11213, USA; yinmon.myat@obhny.org; 2 Comprehensive Cancer Centers of Nevada, Las Vegas, NV 11213, USA; 3 Kirk Kerkorian School of Medicine, University of Nevada, Las Vegas (UNLV), Las Vegas, NV 11213, USA; 4 Department of Medicine, University Nevada, Henderson, NV 11213, USA; 5 Independent Researcher, Nashville, TN 37235, USA; 6 Cleveland Clinic Taussig Cancer Institute, Cleveland, OH 44195, USA; 7 Division of Hematology & Oncology, Department of Medicine, Vanderbilt University Medical Center, Nashville, TN 37235, USA; 8 Vanderbilt-Ingram Cancer Center, Nashville, TN 37235, USA

**Keywords:** rare cancers, tumor-agnostic therapy, biomarker, trial design, precision oncology, early-phase clinical trials, cellular immunotherapy, antibody-drug conjugates, cellular therapy, targeted therapy, fusion inhibitors, translational medicine

## Abstract

Rare cancers represent a substantial health burden, comprising 25% of cancer mortality. Early-phase oncology trials are essential for rare cancers, where limited patient numbers, biological heterogeneity, and the absence of established standards of care necessitate innovative approaches to drug development. Advances in trial design—including basket, umbrella, and adaptive platforms—have enabled efficient evaluation of molecularly defined subgroups and accelerated identification of clinically meaningful activity across rare tumor types. Early-phase studies have demonstrated the therapeutic potential of antibody-drug conjugates, immunotherapy, targeted agents, and cellular therapies, particularly when guided by predictive biomarkers and tumor-agnostic strategies. These approaches have expanded treatment options for patients with historically limited therapeutic opportunities. Persistent challenges remain, including restricted trial access, difficulties with patient accrual, variability in biomarker assessment, and uncertainty regarding generalizability of outcomes. Additional challenges persist after early-phase trial completion, as continued access to therapies demonstrating clinical benefit may remain limited when regulatory approval or broader clinical availability has not yet been established. Evolving regulatory and collaborative frameworks have partially mitigated these barriers by supporting multi-institutional enrollment and pathway-flexible approvals. Over the past decade, biomarker-driven strategies have been increasingly incorporated into modern clinical trial design, shifting the field away from traditional histology-based approaches. Continued innovation in trial design, biomarker integration, and collaborative infrastructure will be essential to sustaining progress and expanding durable clinical benefit for patients with rare cancers.

## Simple Summary

Rare cancers account for a significant proportion of cancer-related deaths, but often lack effective treatment options because of limited patient numbers, biological diversity, and the absence of established standards of care. Over the past decade, oncology clinical trials have increasingly shifted from traditional histology-based approaches toward biomarker-driven and tumor-agnostic strategies. Early-phase clinical trials now frequently use innovative designs, including basket, umbrella, and adaptive trials, to evaluate therapies in molecularly defined patient populations more efficiently. These approaches have expanded treatment opportunities through targeted therapies, immunotherapy, antibody-drug conjugates, and emerging cellular therapies guided by predictive biomarkers. Despite this progress, challenges such as limited trial access, difficulties with patient enrollment, and variability in biomarker testing remain significant barriers. Continued advances in precision oncology, collaborative trial networks, and biomarker integration will be essential to improving outcomes for patients with rare cancers.

## 1. Introduction

### Defining Rare Cancers and Their Collective Mortality Burden

Rare cancers are an important avenue in oncology due to their overall burden on cancer mortality. Rare cancers and their definition vary in different regions due to differing disease patterns. The National Cancer Institute (NCI) defines it as any cancer type with an incidence of ≤15 cases per 100,000 people per year in the United States [1]. However, European guidelines adopt a stricter definition with ≤6 new cases per 100,000 persons per year.

Rare cancers have distinct diagnosis patterns and are further categorized using the International Classification of Diseases for Oncology (ICD-O) into hierarchical tiers based on morphology and topography, with Tier 1 encompassing major organizationally relevant groups and Tier 2 including clinically significant entities with consistent diagnostic and therapeutic approaches [2]. Incidence is preferred over prevalence as a metric because low-incidence cancers with longer survival may appear more common, whereas common cancers with poor survival may appear rarer than they are [3].

Rare cancers represent a major global oncology burden despite their individual rarity. Based on the International Agency for Research on Cancer (IARC) and World Health Organization (WHO), rare cancers constitute 25–30% of all cancer diagnoses and carry worse long-term outcomes compared to more common tumors [4,5]. Despite their collective mortality, treatments are often delayed. Yang and colleagues found that more than one third of patients did not initiate treatment within 30 days of diagnosis [1]. In the United States, 21% of invasive cancers are classified as rare, with 59% of rare solid tumors diagnosed at regional or distant stages compared to 45% of common cancers [2,6]. Rare cancers comprise 25% of cancer-related deaths; however, projections suggest substantial increases by 2050, particularly for bladder and pancreatic cancers [5,7]. These disparities often result in diagnostic delays and limited access to specialized centers. Furthermore, limited treatment options and less research funding exacerbate the unmet needs in this population. Pharmaceutical investment in rare cancer drug development may also be constrained by financial challenges associated with small patient populations and limited commercial incentives, which can hinder large-scale clinical development [8]. These challenges contribute to poor outcomes observed in rare cancers.

Given these challenges, early-phase (Phase I/II) clinical trials are a critical avenue for advancing therapies in rare cancers. These trials serve as the primary platform for signal-seeking studies, biomarker identification, and accelerated approval pathways, offering a feasible route to new treatments for small, underserved patient populations. However, access to early-phase trials often requires referral to specialized academic centers, creating additional geographic and logistical barriers for many patients [9]. Understanding the progress and evolving strategies in early-phase drug development is therefore essential to addressing the global burden of rare cancers.

This narrative review examines early-phase drug development in rare cancers, emphasizing studies from the past decade while retaining earlier landmark trials. PubMed/MEDLINE and Google Scholar were searched through March 2026 using combinations of “rare cancer,” “early-phase trial,” “basket trial,” “umbrella trial,” “adaptive trial,” “tumor-agnostic,” and “biomarker,” together with terms for selected molecular targets and therapies. Reference lists, ClinicalTrials.gov, and regulatory sources were also reviewed; U.S. Food and Drug Administration (FDA) approval statuses and guidance were checked through September 2026. Original trial reports and regulatory documents were prioritized for clinical and approval claims. This was a purposive narrative review: studies were not selected under a systematic-review protocol, and no formal risk-of-bias assessment or meta-analysis was conducted. Cross-study comparisons therefore require caution because populations, assays, endpoints, and follow-up differ.

## 2. Trends in Early-Phase Rare Cancer Trials

**Figure 1:**
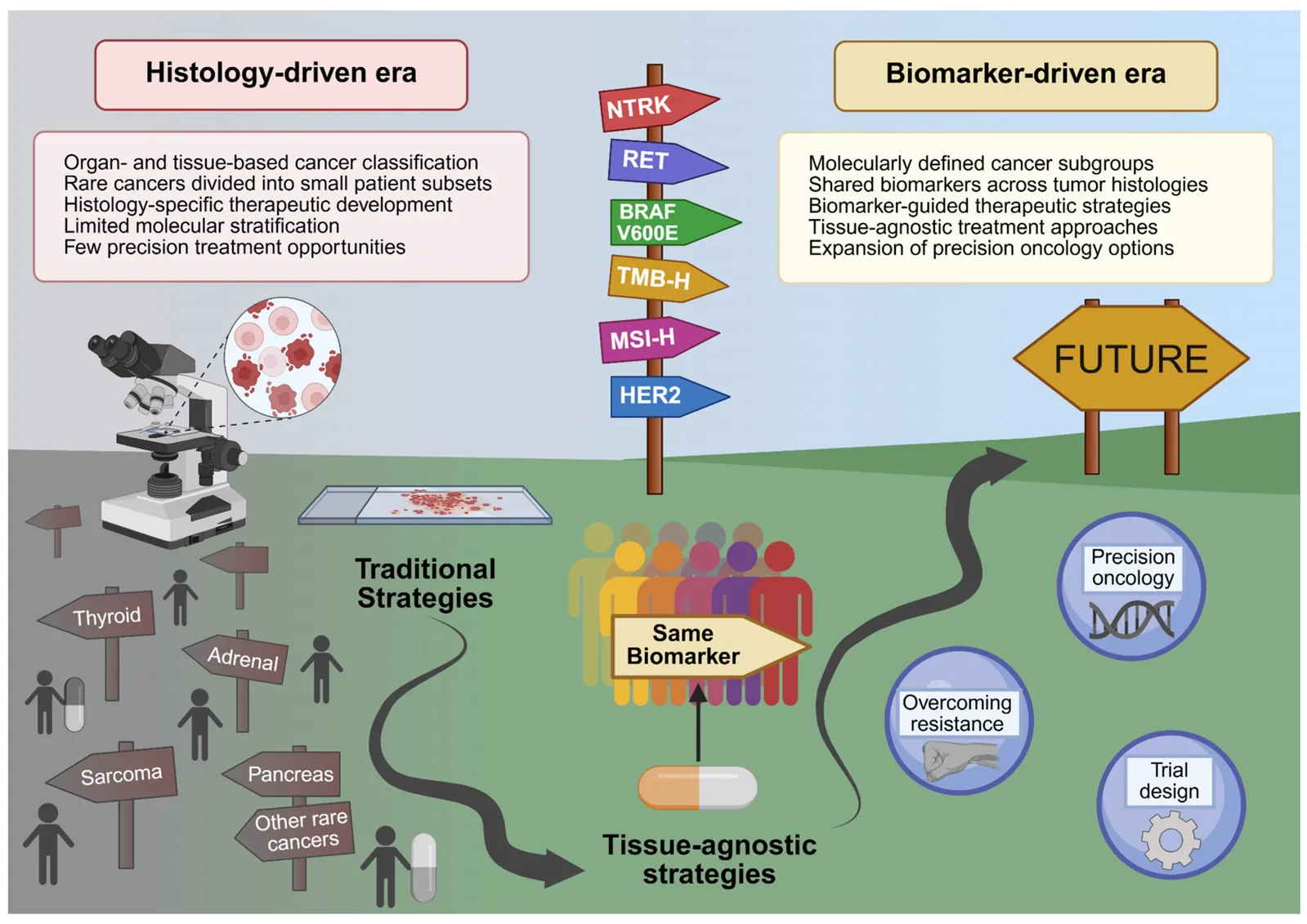
Conceptual schematic illustrating the macro-level evolution from traditional histology-driven to biomarker-driven strategies.

Traditional randomized controlled trials (RCTs) remain largely not feasible in rare cancers due to small, geographically dispersed patient populations, disease heterogeneity, the lack of validated genomic targets and lack of standardized therapeutic approaches. Over the past decade, early phase clinical trials for rare cancers have increasingly shifted towards master protocol frameworks, including basket, umbrella and adaptive trials. These innovative designs have allowed for a more biomarker-driven approach. Historically, clinical trials involved targeting cancer histology types. However, this approach is particularly difficult for rare cancers where clinical and histological clones and subclones are more heterogeneous. There has been increasing adoption of tumor-agnostic approaches, in which targeted therapies and other novel agents are directed against actionable molecular alterations regardless of tumor histology or rare cancer subtype (Figure 1). Despite the promise of pan-cancer strategies, tissue-agnostic efficacy may still vary substantially across tumor lineages, even among tumors harboring the same biomarker. This shift toward agnostic treatment paradigms reflects the broader integration of precision oncology strategies, with comprehensive genomic profiling and molecular prescreening programs now central to early-phase clinical trial design. Modern approaches also include the expanding use of liquid biopsies and A.I-assisted molecular profiling in early-phase rare cancer trials [10]. Actionable mutations such as BRAF V600E, Neurotrophic Tyrosine Receptor Kinase (NTRK) fusions, and Rearranged During Transfection (RET) alterations can be rapidly identified, facilitating earlier and more personalized intervention, particularly for rare cancer types with limited therapeutic options. Tumor mutational burden-high (TMB-H) is another emerging tumor-agnostic marker which is commonly defined as 10 mutations per megabase (mut/Mb) or higher. The incidence of TMB-H had an incidence of approximately 12.8% in less common cancers such as biliary tract cancers and mesothelioma [11]. Similarly, immunotherapies targeting deficient mismatch repair (dMMR) and microsatellite instability-high (MSI-H) biomarkers are integral for advancement of management of rare cancers. The integration of precision medicine has also facilitated the development and testing of novel therapeutics such as antibody-drug conjugates (ADCs), expanding the range of investigational treatments available for these underserved populations.

Over this period, the number of rare cancer trials has grown substantially, with increased use of adaptive designs, patient engagement strategies, and global trial networks. For instance, analysis of the GlobalData database demonstrated that industry-sponsored basket trials increased from 10 to 40 between 2016 and 2021, while by 2021 there were approximately 42 basket trials and seven umbrella trials, contributing to a total of over 5000 trials for rare cancers initiated that year [12]. Despite this growth, these innovative designs remain underutilized, and significant gaps remain in understanding the clinical outcomes of rare cancer populations due to ongoing recruitment challenges, disease complexity, and limited population sizes. Despite tumor-agnostic indication, modern therapies have varied efficacy and survival data likely due to tissue-specific factors. Real-world data show that rare alterations, such as NTRK and RET fusions (<0.5% of tumors), have low uptake despite high efficacy, and outcomes vary across tumor types. Notably, patients with non-trial tumor types that were underrepresented or omitted from pivotal trials experience similar benefits to trial-included lineages, supporting broader applicability. These findings highlight both the promise and limitations of tissue-agnostic therapies and underscore the need for increased adoption, larger cohorts, and longer follow-up [13].

Although Phase I trials remain foundational to rare cancer drug development, recent years have seen a relative increase in Phase II studies, reflecting maturation of early-phase pipelines rather than a shift away from exploratory research [14]. This trend suggests that biomarker-driven and adaptive Phase I/II designs are increasingly generating actionable efficacy signals, enabling expansion into Phase II signal-seeking cohorts.

Over the last decade, rare cancer trials have transitioned from fragmented, histology-limited, underpowered studies to biomarker-driven, adaptive, and translationally rich early-phase programs. While significant challenges persist—particularly in recruitment, heterogeneity, and equitable access—the field has demonstrated that innovative trial design and precision medicine can fundamentally reshape drug development for rare cancers.

This conceptual figure illustrates the evolution of oncology from traditional histology-driven treatment approaches toward biomarker-driven and tumor-agnostic precision oncology. In the histology-driven era (left), patients are categorized according to tumor site of origin, reflecting conventional organ-based classification and treatment strategies. The directional arrow represents the transition toward biomarker-driven care (center), in which patients are stratified based on shared molecular alterations rather than tissue histology. A single targeted therapeutic agent directed against a common biomarker highlights the principle of molecularly guided treatment across multiple tumor types. The central panel depicts a growing spectrum of actionable biomarkers, emphasizing the increasing molecular complexity and refinement of modern precision oncology. This progression ultimately leads to strategies involving future precision oncology, modern clinical trial designs, and methods to overcome resistance mechanisms. This macro-level schematic omits specific genomic signaling pathways, intracellular feedback loops, and individual drug pharmacokinetic profiles to maintain a high-level conceptual framework. Created in BioRender. Myat, Y. (2026) https://BioRender.com/u7yi5bm.

## 3. Impact of Innovative Trial Designs

**Figure 2:**
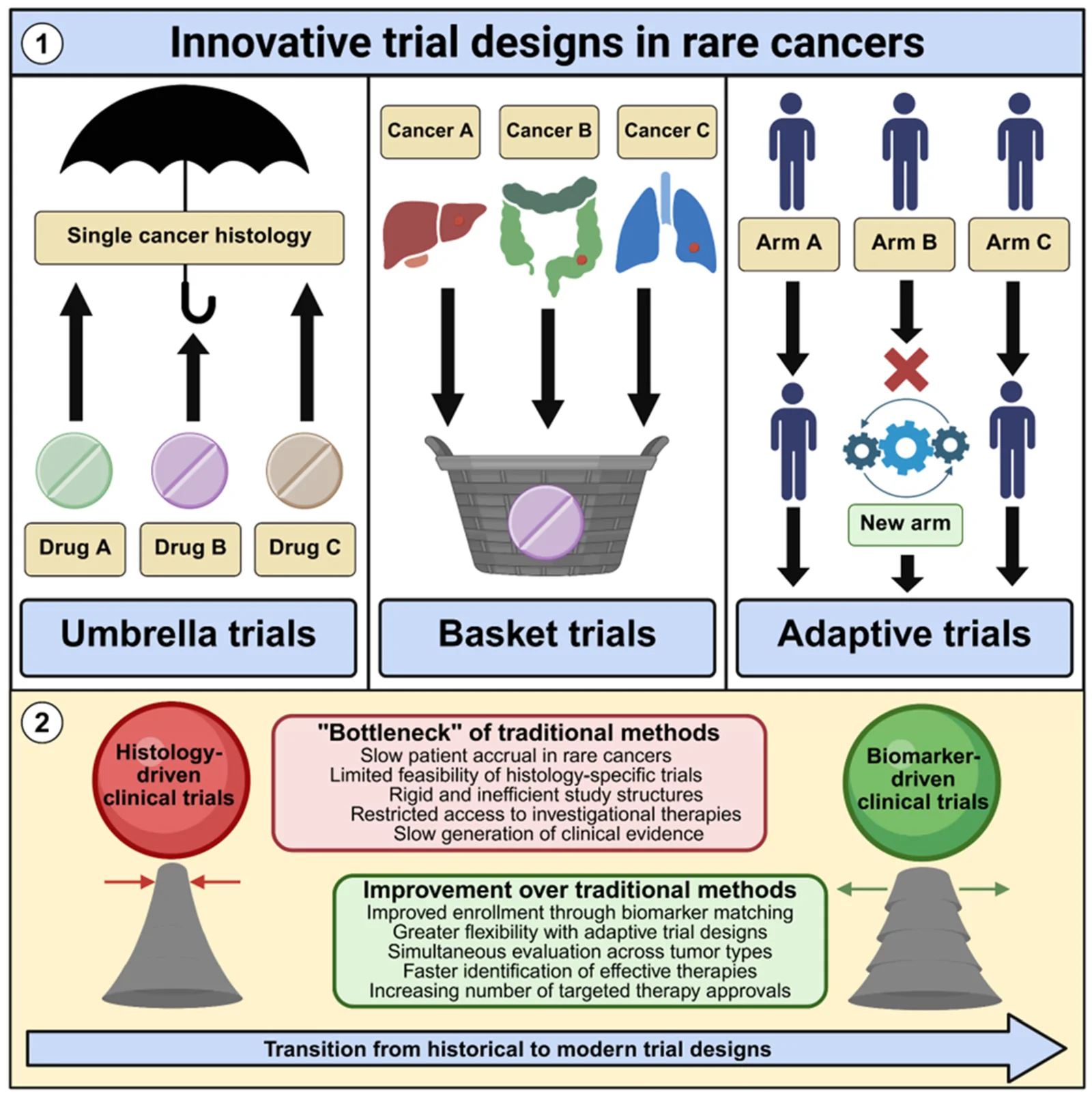
Innovative Trial Designs in Rare Cancers.

The increasing use of innovative trial designs, collectively known as master protocols, including basket trials, umbrella trials, and adaptive trials, has played a key role in advancing early-phase research in rare cancers (Figure 2). These designs provide a framework that enables the concurrent evaluation of multiple therapeutic agents, biomarkers, or disease subtypes, helping to overcome the logistical and geographic limitations of traditional trial designs. Programs such as Project Orbis have also allowed for global collaboration for clinical trials leading to quicker approval of oncology therapies. Beyond improving operational efficiency and patient accrual, master protocols can generate timely evidence that supports accelerated regulatory pathways, facilitating earlier access to therapies in rare cancers.

### 3.1. Basket Trial Design

Basket trials enroll patients across multiple tumor histologies harboring a shared molecular alteration, enabling the simultaneous evaluation of a single therapy or therapeutic class across diverse cancer types. These designs have been foundational to the development of modern tumor-agnostic approvals and are particularly valuable in rare cancers, where histology-specific trials are often infeasible due to limited patient populations. By aggregating patients across tumor types, basket trials enable the study of molecularly defined rare populations that would otherwise remain understudied and have been instrumental in tumor-agnostic drug development, particularly for therapies targeting strong, actionable biomarkers such as NTRK fusions and MSI-H status. Notable examples include the *MyPathway* trial, which enrolled multiple tumor types harboring HER2 amplification, and the Rare Oncology Agnostic Research (ROAR) trial, which targeted BRAF V600E mutations across rare tumors. The *NCI-MATCH* study exemplifies a large-scale basket design with multiple tumor-agnostic arms targeting actionable mutations across diverse cancer types [14,15,16,17,18]. Basket trials facilitate efficient patient accrual, generate early efficacy signals, and have supported tissue-agnostic FDA approvals, providing therapeutic options to patients with otherwise limited treatments. Challenges include tumor heterogeneity diluting efficacy signals, small subgroup sample sizes, and regulatory uncertainty [12]. Responses to the same biomarker-driven therapy may still vary across histologies due to lineage-specific biology and tumor microenvironment differences. Furthermore, these trials demonstrate how molecular profiling can guide treatment across histologies, highlighting the transformative potential of precision medicine in rare cancers [19].

### 3.2. Umbrella Trial Design

Umbrella trials focus on a single histologic cancer type while testing multiple targeted therapies across distinct molecular subgroups. Patients are assigned to treatment arms based on genomic or molecular alterations, enabling parallel evaluation of several targeted strategies within one disease context. These designs also allow for adaptive modification, including the addition or closure of treatment arms as emerging molecular data evolve [20]. In rare cancers, where molecular heterogeneity is common, umbrella designs provide an efficient mechanism to test multiple hypotheses without requiring separate trials, thereby accelerating discovery of effective therapies. The *Lung-MAP* trial, which incorporated multiple genomically defined substudies in non-small-cell lung cancer, exemplifies this approach [15,21]. Umbrella trials improve understanding of molecular subtypes, enable precision-matched therapy assignment, and contribute to more informed regulatory decision-making. Successful implementation of umbrella trials requires robust, comprehensive genomic screening infrastructure to enable accurate and timely molecular characterization of patients for appropriate treatment assignment [22]. Limitations include the need for sufficient molecular diversity within a tumor type and small cohort sizes for rare alterations, which may impact interpretability.

### 3.3. Adaptive Trial Design

Adaptive trials allow pre-planned modifications to study elements—including randomization ratios, sample size, or treatment arms—based on interim analyses of accumulating safety and efficacy data. These designs are particularly beneficial in rare cancers, where patient numbers are limited and ethical considerations favor minimizing exposure to ineffective therapies. Adaptive designs can accelerate identification of promising agents, improve patient allocation, and shorten development timelines, often offering cost-saving benefits [23]. Phase I/II designs are being increasingly used in biomarker-driven oncology trials. This approach reduces the number of patients needed, accelerates dose finding, and minimizes patient exposure to sub-therapeutic or excessively toxic doses [24,25]. The study may also be stopped early for responses exceeding a predefined target early in the trial. Notably, patients with rare cancers often show poorer overall survival than common cancers, yet may demonstrate some of the highest response rates to targeted therapies [26]. The *I-SPY 2* trial in breast cancer exemplifies adaptive randomization based on interim results [15]. Many adaptive trials employ Bayesian statistical frameworks and response-adaptive methods, although limited historical data, operational complexity, and regulatory concerns can pose challenges. Careful pre-specification of adaptation rules and extensive simulation are essential to maintain trial integrity. Adaptive trials may further support accelerated regulatory pathways by generating timely and flexible evidence, which can facilitate collaborative review frameworks such as Project Orbis [27]. These designs may be particularly valuable for rapidly evolving precision oncology landscapes and emerging biomarkers.

### 3.4. Statistical and Operational Challenges of Clinical Trial Design

Basket and umbrella trials share screening infrastructure, but each substudy still needs a prespecified population, endpoint, comparison, and decision rule. A pooled response rate can conceal a resistant histology; a tiny cohort can also give an unstable estimate. Borrowing information across cohorts may improve precision when effects are genuinely similar, but can mislead when lineage modifies treatment response. Simulations should assess these possibilities before borrowing or adaptive rules are used [28].

Multiplicity depends on the clinical questions. Cohort-level two-stage designs can control false-positive conclusions within a specified cohort. A trial-wide adjustment is not automatically required across independent drugs or diseases: FDA’s June 2026 draft master-protocol guidance generally does not recommend it across distinct clinical objectives. Multiple endpoints, doses, and interim decisions within an individual drug evaluation still need appropriate error control and assessment of operating characteristics [29,30]. Adaptive trials must prespecify stopping, allocation, and sample-size rules and examine their performance under plausible enrollment and treatment-effect scenarios.

Finally, an objective response in a small single-arm cohort demonstrates activity, not a comparative survival benefit. Interpretation should include confidence intervals, response duration, toxicity, and the feasibility of confirming clinical benefit [30]. Central testing, tissue requirements, assay turnaround, and trial costs can further limit enrollment and generalizability.

Furthermore, high-profile clinical trials can falter due to the operational and biological limits of master protocols and small single-arm cohorts. Landmark efforts like the negative randomized SHIVA trial, underwhelming NCI-MATCH substudies, and variable immunotherapy responses in SARC028 illustrate that targeted therapies often fail across disparate lineages due to complex signaling networks and distinct microenvironments [31,32,33]. Challenges may particularly be present for ultra-rare indications. Additionally, high screening-to-enrollment attrition, early arm closures, and masked inter-cohort heterogeneity emphasize the need for prespecified futility boundaries, realistic histology-specific benchmarks, and cautious interpretation of pooled cross-histology data [20].

Collectively, basket, umbrella, and adaptive trial designs have transformed early-phase rare cancer research by enabling more efficient evaluation of targeted therapies, facilitating earlier patient access to novel treatments, and supporting regulatory approvals for cancers with limited prior options. Beyond operational efficiency, these designs have advanced the integration of precision medicine into clinical practice, offering actionable treatment strategies for patients who previously had few therapeutic avenues. In settings where even these approaches are infeasible due to extreme rarity, N-of-1 trials provide a complementary framework to systematically assess molecularly matched therapies at the individual level and identify exceptional responses that can inform future drug development. As these approaches continue to evolve, they are expected to further accelerate the development of targeted and tumor-agnostic therapies in rare cancers.

This conceptual figure provides a simplified overview of innovative clinical trial designs. Panel 1 illustrates the three major trial designs: umbrella, basket, and adaptive trials. Umbrella trials evaluate multiple treatment regimens within a single cancer histology based on distinct molecular alterations. Basket trials assess a single targeted therapy across multiple cancer histologies sharing a common biomarker. Adaptive trials evaluate multiple treatment arms simultaneously, with ongoing modifications based on emerging data, including discontinuation of ineffective arms and incorporation of new interventions. Panel 2 highlights the evolution of oncology clinical trials from traditional histology-driven approaches to biomarker-driven precision oncology. Conventional histology-based trial models were often limited by small patient populations, low trial enrollment, rigid study structures, and a scarcity of approved therapies for rare cancers. The adoption of innovative trial designs and biomarker-guided strategies has improved trial efficiency, flexibility, and patient enrollment, ultimately contributing to the expanding number of therapeutic options available for rare cancers. This simplified diagram omits granular statistical powering formulas, complex Bayesian interim analysis rules, and specific regulatory protocol amendments required for trial execution. Created in BioRender. Myat, Y. (2026) https://BioRender.com/3mhlhts.

## 4. Emerging Therapies

Emerging therapies are particularly important in rare cancers, where standard treatment options are limited and outcomes are often poor. By targeting specific molecular alterations, gene fusions, or modulating the immune system, these approaches enable precision oncology. Tumor-agnostic treatments further extend therapeutic options for patients with rare malignancies, offering the potential for durable responses and addressing critical unmet medical needs (Table 1a–c).

### 4.1. Immunotherapy

Immunotherapy has transformed cancer treatment by harnessing the immune system to target tumors, offering new options for patients with rare cancers who often have limited therapies (Table 1a). Pembrolizumab was the first FDA-approved tumor-agnostic therapy, receiving accelerated approval for advanced dMMR/MSI-H solid tumors in the refractory setting, including colorectal cancer (CRC), based on five phase II trials [34,35,36,54]. Dostarlimab, another PD-1 inhibitor, was initially approved for dMMR endometrial cancer, though limited data exist in rare cancers [37,38,55]. Pembrolizumab also received accelerated approval for tumor mutational burden-high (TMB-H) solid tumors in 2020 for a pan-cancer cohort. TMB-H was defined by the FDA as ≥10 mutations per megabase (mut/Mb), although this cutoff may vary throughout the literature [39,56]. More recently, immune checkpoint inhibitor retifanlimab-dlwr (Zynyz) was approved for locally recurrent or metastatic squamous cell carcinoma of the anal canal [57,58] and penpulimab-kcqx was approved for non-keratinizing nasopharyngeal carcinoma [59]. Combination immunotherapy regimens, including dual cytotoxic T-lymphocyte antigen 4 (CTLA-4) and PD-1 inhibition, have also seen increasing use (Table 1a) [40]. Responses to immunotherapy in rare cancers are often heterogeneous across tumor types, reflecting underlying differences in tumor microenvironment, lineage-specific biology, and biomarker expression profiles [60].

In early-phase rare cancer trials, bispecific antibody (BsAb) development has shifted toward next-generation formats designed to improve safety, pharmacokinetics, and target specificity in small, molecularly defined populations. Contemporary phase I studies increasingly evaluate affinity-tuned T-cell engagers, conditionally activated BsAbs, and multi-specific constructs that aim to mitigate systemic toxicity while addressing heterogeneous antigen expression. These advances align well with basket and biomarker-driven trial designs, enabling efficient signal detection and dose optimization in rare cancers where traditional escalation strategies and large cohorts are not feasible. Blinatumomab (CD19 × CD3), the first BsAb to receive FDA approval, demonstrated clinical efficacy in relapsed/refractory B-cell precursor acute lymphoblastic leukemia and in minimal residual disease-positive disease. Several next-generation BsAbs are being evaluated, including zenocutuzumab targeting HER2 × HER3, highlighting the expanding therapeutic potential of this class [47,48,61]. The immunotherapy landscape is further diversified by next-generation cellular strategies, such as chimeric antigen receptor (CAR) T-cell, T-cell receptor (TCR), and natural killer (NK) cell therapies; these approaches offer a complementary mechanism of action that is increasingly relevant for addressing the unique antigenic profiles of rare cancers [62].

### 4.2. Targeted Therapies

Targeted therapies exploit specific genetic or molecular alterations driving tumor growth, offering precision treatment options for patients with rare cancers who often have limited standard therapies. By identifying actionable mutations or pathways, these therapies enable personalized approaches that can improve efficacy and reduce off-target toxicity [41]. Dabrafenib and trametinib represent the first BRAF inhibitor combination approved for tumor-agnostic use (Table 1b). Importantly, CRC remains a notable exception to this tissue-agnostic approval. In CRC, single-agent BRAF blockade triggers rapid, adaptive epidermal growth factor receptor (EGFR)-mediated feedback loops that reactivate the MAPK pathway, causing intrinsic resistance [41]. This includes on-target and off-target mechanisms which may bypass inhibition and result in hyperproliferation of tumor cells. Such pathway bypass mechanisms underscore the limitations of targeted monotherapies, particularly in ultra-rare cohorts with limited longitudinal data. Consequently, vertical combination regimens—such as dual BRAF and EGFR inhibition—and next-generation inhibitors are critical to overcoming acquired resistance mechanisms [63]. Additionally, KRAS inhibitors are rapidly emerging as a therapeutic option, particularly in pancreatic cancer, and may eventually gain tumor-agnostic approval for biomarker-selected rare cancers; however, no regulatory approval for tumor-agnostic indications currently exists. Other targets involved in cellular proliferation include fibroblast growth factor receptor (FGFR), dysregulation of which drives aberrant cancer cell growth. Erdafitinib is an example of an FGFR inhibitor that has shown response across different histologies; however, no FDA approval for tumor-agnostic use is available yet (Table 1b) [44,45,46,64].

Other recent approvals for rare cancers include the HIF-2α-targeted agent belzutifan (Welireg) for patients 12 years and older with locally advanced, unresectable, or metastatic pheochromocytoma or paraganglioma [65], and the combined regimen of avutometinib and defactinib for low-grade serous ovarian cancer [66]. These approvals highlight a shift toward targeting rare biomarkers beyond the more common KRAS G12C mutations, possibly expanding therapeutic options for rare malignancies.

### 4.3. Fusion Inhibitors

The clinical integration of fusion inhibitors stands as one of the most definitive paradigms of successful biomarker-driven drug development in rare cancers, illustrating how molecularly defined cohorts can achieve robust responses regardless of histological origin. These oncogenic fusions—notably those involving NTRK and RET—act as potent, tissue-agnostic drivers that transcend traditional diagnostic boundaries, offering a high-yield therapeutic target within rare and molecularly distinct subsets [67].

Larotrectinib and entrectinib, both NTRK inhibitors, as well as selpercatinib, a RET inhibitor, are approved for tumor-agnostic use (Table 1c). Larotrectinib was approved for refractory NTRK fusion-positive solid tumors in adults and children in November 2018 and later received full FDA approval in April 2025 [49]. Entrectinib, which also targets ROS1 and ALK, was approved on 15 August 2019 for NTRK fusion-positive solid tumors in patients ≥ 12 years [50]. Selpercatinib received approval for advanced or metastatic RET fusion-positive solid tumors on 21 September 2022, following the global phase I/II LIBRETTO-001 trial [51,68].

Most recently, repotrectinib received accelerated FDA approval for advanced or metastatic *NTRK* fusion-positive solid tumors following the TRIDENT-1 trial (Table 1c) [52,53]. As a next-generation macrocyclic tyrosine kinase inhibitor (TKI) designed to overcome gatekeeper mutations, repotrectinib demonstrated an objective response rate (ORR) of 58% in TKI-naïve and 50% in TKI-pretreated patients, notably maintaining efficacy in the presence of CNS metastases [69].

Furthermore, central nervous system (CNS) penetration has emerged as a critical benchmark for next-generation fusion inhibitors, particularly in malignancies driven by ALK, ROS1, and NTRK alterations [70]. For instance, in patients with NTRK fusion-positive solid tumors with baseline CNS metastasis, entrectinib demonstrated durable intracranial efficacy [71]. Repotrectinib also maintained efficacy in the presence of CNS metastasis [69].

Although first-generation fusion inhibitors have demonstrated durable clinical efficacy, tumors inevitably develop resistance and become refractory. In *NTRK* fusion-positive malignancies, resistance is primarily driven by acquired mutations within the NTRK kinase domain, such as solvent-front substitutions. Next-generation TRK inhibitors—including selitrectinib and repotrectinib—were specifically designed with a compact binding interface to bypass this steric hindrance and restore therapeutic control [72]. Similarly, in *RET* fusion-driven cohorts, acquired solvent-front mutations account for 20% to 25% of treatment failures [73]. To counter these on-target escape mechanisms, next-generation RET inhibitors are actively being deployed to overcome steric barriers and salvage durable responses.

### 4.4. Antibody-Drug Conjugates (ADCs)

ADCs are targeted cancer therapies that combine tumor specific antibodies with a cytotoxic drug, allowing selective delivery of the drug to cancer cells while minimizing systemic toxicity. The monoclonal antibody binds to a tumor-associated antigen, triggering internalization and enabling targeted intracellular delivery of the cytotoxic payload. Linker design is critical, as it must ensure systemic stability to minimize off-target toxicity while allowing efficient drug release within tumor cells. Each component of an ADC contributes to its overall therapeutic performance, and even subtle structural modifications can significantly alter in vivo behavior [74]. Early-generation ADCs demonstrated limited clinical efficacy, largely attributed to unstable linkers and suboptimal payload potency. For example, gemtuzumab ozogamicin, when combined with chemotherapy, failed to improve survival or toxicity outcomes compared with chemotherapy alone, ultimately leading to its withdrawal from the market [75]. Collectively, antigen selection, linker stability, payload potency, and internalization efficiency are key determinants of ADC activity.

Since then, the ADC role in the treatment of rare cancers has expanded after improvements in potency of the cytotoxic drug. In the DESTINY-PanTumor02 trial, trastuzumab-deruxtecan (T-DXd) demonstrated efficacy across seven tumor types, including tumors of the reproductive system (endometrial, cervical and ovarian), bladder, biliary tract, and pancreas (Table 1b). In cohort 7, metastatic or advanced rare tumors with HER2 overexpression were also included. Overall, 37.1% of patients had a response with median PFS of 6.9 months and median OS of 13.4 months [42,43]. Although responses are durable, interstitial lung disease and pneumonitis are well-described and serious side effects [76].

Resistance to antibody-drug conjugates (ADCs) involves multiple mechanisms, including alterations in antigen–antibody binding, antigen expression changes, payload resistance, lysosomal dysfunction, and impaired intracellular trafficking. Disruption of antigen–antibody binding may arise from tumor heterogeneity, including low, heterogeneous, mutated, or lost target antigen expression, as has been described for HER2 expression variability [77]. A bystander cytotoxic effect may also occur, where the released payload diffuses into neighboring tumor cells with lower or absent target expression. While this mechanism may contribute to off-target toxicity, it has also been proposed as a strategy to overcome intratumoral heterogeneity. Consistent with this, clinical activity has been observed in HER2-low and HER2-ultralow cancers, supporting the broader potential of ADCs in tumor-agnostic and rare cancer settings [78].

The therapeutic landscape for rare malignancies is rapidly evolving through the use of next-generation ADCs that target novel surface antigens. A notable example is pivekimab sunirine targeting CD123 which has demonstrated efficacy in patients with blastic plasmacytoid dendritic cell neoplasm [79]. Furthermore, risvutatug rezetecan, a B7-H3-targeted ADC, received Orphan Drug Designation in March 2026 for small-cell lung cancer—a designation supported by promising clinical data that suggests broad potential across other difficult-to-treat solid tumors [80,81]. There is also a growing interest in combinations of ADC with immunotherapy, particularly CTLA-4 and PD-L1 inhibitors, for treatment of rare cancers (Table 1a). This is likely due to opportunities to overcome resistance and early-phase trials showing promising results. Combinations include trastuzumab emtansine (T-DM1) and atezolizumab, T-DM1 and pembrolizumab, as well as T-DXd with durvalumab. These combinations may aid in treatment-refractory cancers due to factors inhibiting immunotherapy such as immunosuppressive microenvironments and high tumor heterogeneity [82].

### 4.5. Cellular Therapies

Adoptive cellular therapy (ACT) is an evolving treatment modality in rare cancers that harnesses immune effector cells to selectively target tumor-associated antigens. It consists mainly of CAR T-cell therapies, tumor-infiltrating lymphocyte (TIL) therapies, and TCR therapies. Historically, ACT has had efficacy in hematological malignancies compared to solid tumors. This could be due to various reasons such as difficulty targeting and unfavorable tumor microenvironments (TME) [83]. However, advances in genetic engineering have expanded these approaches beyond hematologic malignancies into solid and molecularly defined rare tumors.

CAR T-cell therapies are being adapted from hematologic malignancies to solid and molecularly defined rare tumors, with early-phase investigations ongoing in rare cancers. In particular, GD2, mesothelin, and CLDN18.2 are being evaluated as targets in rare solid tumors, with GD2 demonstrating early clinical activity in neuroblastoma [84], mesothelin showing feasibility in mesothelioma [85], and CLDN18.2 supported by prior clinical validation with agents such as zolbetuximab [86], although no CAR-T therapies have yet achieved regulatory approval. Trials aim to overcome challenges related to tumor heterogeneity, antigen escape, and the immunosuppressive tumor microenvironment [87].

In contrast, a major milestone was achieved with the FDA approval of Amtagvi in early 2024 for unresectable or metastatic melanoma [88], marking the first TIL therapy approved for a solid tumor. TIL therapy is being investigated in selected immunogenic solid tumors beyond melanoma, including cervical cancer, head and neck squamous cell carcinoma, and sarcomas, with early-phase studies demonstrating feasibility and preliminary antitumor activity following adoptive transfer of ex vivo expanded autologous T cells [89].

Next-generation TCR-based approaches, including TCR–CD3 bispecifics (ImmTACs), further expand the ability to target intracellular antigens. Tebentafusp represents a landmark example, receiving FDA approval in January 2022 for HLA-A*02:01-positive unresectable or metastatic uveal melanoma based on the phase 3 IMCgp100-202 trial [90]. In parallel, TCR-engineered T cell therapies (TCR-T) enable ex vivo modification of autologous T cells to recognize intracellular tumor antigens in an HLA-restricted manner. Afamitresgene autoleucel (afami-cel; Tecelra) targets the cancer-testis antigen MAGE-A4 and was the first FDA-approved TCR gene therapy for adults with unresectable or metastatic synovial sarcoma after prior chemotherapy [91].

Rare solid tumors present biological and access barriers to cellular therapy. Variable antigen expression, limited T-cell trafficking, dense stroma, and immunosuppression can reduce activity, while expression in healthy tissue can cause toxicity [92]. Small biopsies complicate target validation. Autologous manufacturing adds collection, production time, bridging treatment, travel, specialized monitoring, and substantial costs. Trials should report screening-to-infusion attrition and manufacturing failures alongside outcomes among infused patients. Proposed dual-target or off-the-shelf approaches remain investigational in these settings [93]. To counter these formidable barriers, next-generation engineered cellular platforms—such as dual-targeting CAR T-cell treatments to prevent antigen escape, ‘armored’ CARs that secrete transgenic cytokines to remodel the microenvironment, and switchable systems that provide precise control over toxicity—are being actively developed to enhance therapeutic persistence and safety [94]. In parallel, NK-cell and CAR-NK therapies have emerged as promising “off-the-shelf” cellular platforms with potentially favorable safety profiles and are increasingly being evaluated in early-phase trials for rare cancers [95]. Oncolytic viral therapies, such as Talimogene laherparepvec, further complement these approaches by promoting immune activation through direct tumor lysis and are being investigated alone or in combination strategies in rare tumors [96]. Collectively, these advances highlight the feasibility, potential, and expanding diversity of cellular immunotherapies in the treatment of rare cancers.

## 5. Biomarker-Driven Precision Approaches in Rare Tumors

In rare cancers, precision oncology is increasingly shifting from a histology-based framework toward biomarker-driven patient selection in early-phase clinical trials. Biomarkers are routinely integrated into trial design to identify actionable alterations, enrich for treatment responders, and support the development of targeted therapies and immunotherapies in small, molecularly defined populations (Table 2).

Various biomarkers have led to FDA approvals across solid tumors, expanding therapeutic options for patients with rare cancers and addressing limitations of small patient populations in early-phase, histology-specific trials. These include tumor-agnostic FDA approvals based on predictive biomarkers, such as pembrolizumab (MSI-H/dMMR and TMB-H), dostarlimab (dMMR), dabrafenib plus trametinib (BRAF V600E), larotrectinib (NTRK fusion), entrectinib (NTRK fusion), selpercatinib (RET fusions), and repotrectinib (NTRK fusions) (Table 2) [97]. Other actionable targets include ROS1, ALK, FGFR, and NRG1 fusions [98]. These fusions are enriched in rare tumors, enabling precision therapy through genomic profiling and expanding treatment options for patients with limited standard therapies.

Importantly, biomarkers often interact to influence therapy outcomes. For example, TMB, MSI, and PD-L1 demonstrate complex interplay in predicting immunotherapy response (Table 2) [98]. TMB-H tumors typically respond to immune checkpoint inhibitors regardless of MSI or PD-L1 status [42], though this can vary by tumor type. PD-L1 expression is measured by tumor proportion score or combined positive score, capturing tumor and immune cell contributions [99]. Immune microenvironment may also be considered as composite biomarkers, however remains poorly characterized and underinvestigated in rare cancers [100]. In rare cancers, appreciating the interplay among biomarkers is crucial for selecting patients and optimizing individualized therapy, particularly in heterogeneous and small populations.

HER2 also presents an interesting biomarker since it is evaluated on a continuous spectrum rather than a strict binary threshold. While historically categorized via binary immunohistochemistry (IHC) cutoffs—such as IHC 3+ for traditional approvals—the clinical paradigm has shifted with therapies like trastuzumab deruxtecan, which demonstrate meaningful activity in HER2-low and HER2-ultralow tumors. Recognizing HER2 as a continuous gradient is critical to avoid missing patients with low expression; this is further reinforced by the payload’s bystander effect, whereby membrane-permeable payloads diffuse from HER2-overexpressing cells to kill adjacent tumor cells with lower or absent target expression [101].

Realizing biomarker-driven precision oncology is hindered by systemic diagnostic disparities, including uneven genomic profiling coverage, out-of-pocket costs, and limited molecular infrastructure outside major centers. Furthermore, diagnostic discordance from local testing frequently leads to rare tumor misclassification and trial misassignment. Expanding universal screening, reimbursement models, and decentralized diagnostic networks is vital to bridge these gaps [102].

Emerging precision strategies are increasingly integrating A.I alongside circulating tumor DNA (ctDNA) liquid biopsies. A.I-driven multi-omic platforms facilitate the synthesis of multi-dimensional datasets, thereby enhancing biomarker development within heterogeneous tumor populations. This computational framework is particularly advantageous for characterizing the complex genomic landscapes of rare malignancies [103,104]. Concurrently, liquid biopsies offer a minimally invasive methodology to characterize heterogeneous malignancies. This approach is particularly valuable in rare cancers, where limited tissue availability and small patient populations necessitate more dynamic and scalable biomarker frameworks. Although clinical evidence remains evolving, serial liquid biopsies may potentially help overcome the inherent limitations of conventional, cross-sectional tissue biopsies by capturing both spatial and temporal tumor heterogeneity, enabling the real-time monitoring of adaptive resistance mechanisms and clonal evolution [105,106,107].

In early-phase trials for rare cancers, RWD is emerging as a critical adjunct to conventional trial design, enabling real-world–derived biomarker refinement. By leveraging biomarker-informed synthetic control arms, trial populations can be matched to real-world cohorts, allowing iterative optimization of biomarker-defined subgroups and improving patient selection. RWD and synthetic control arms offer a valuable and practical approach for ultra-rare cancers, where traditional randomized trials are often infeasible. This approach enhances trial efficiency with earlier detection of likely responders and non-responders. However, RWD analyses also reveal translational gaps; notably, despite favorable efficacy and safety profiles, therapies targeting rare tumor-agnostic alterations, such as NTRK fusions, have demonstrated limited real-world uptake [13].

Real-world cohorts can describe natural history and contextualize single-arm results when randomization is impracticable. They are vulnerable to selection bias, unmeasured confounding, missing data, inconsistent response assessment, and changes in treatment over time. Investigators should align eligibility criteria, index dates, biomarkers, and endpoints and prespecify sensitivity analyses. Statistical weighting cannot establish equivalence to a randomized control. The regulatory usefulness of an external comparison depends on its data quality and the full clinical evidence package [108].

Together, these findings underscore both the potential of real-world–derived biomarker refinement to accelerate precision oncology and the challenges of translating biomarker-driven advances into clinical practice for rare cancers. Thus, biomarker expression and therapeutic response may evolve dynamically over time, reinforcing the importance of longitudinal monitoring.

**Table 2:** Biomarker-Driven Precision Strategies in Rare Cancers.

Biomarker and Representative Therapies	Detection Method	Trial Enrichment Strategy	Clinical Relevance in Rare Cancers	Ref.
**MSI-H/dMMR** **(Pembrolizumab** **Dostarlimab)**	**IHC:** Primary screening modality to evaluate MMR protein expression. **PCR**: Locus-specific testing used as a reflex method for indeterminate IHC results. **NGS/ctDNA**: Tissue or plasma panels to confirm MSI status and simultaneously evaluate TMB.	**Protein loss selection**: Prospective enrichment targeting tumors with documented loss of expression in at least one key mismatch repair protein (MLH1, MSH2, MSH6, or PMS2). **Reflex diagnostic algorithm**: IHC followed by PCR for indeterminate results, resolving baseline screening ambiguities to capture true dMMR phenotypes. **Multi-target genomic screening**: Comprehensive NGS profiling to capture hypermutated, high-neoantigen phenotypes across tissue-agnostic basket protocols.	Pembrolizumab was the first FDA-approved tumor-agnostic therapy. High clinical yield in rare GI malignancies (e.g., small bowel adenocarcinoma, cholangiocarcinoma) and endometrial cancers. Provides a durable, chemotherapy-sparing option via anti-PD-(L)1 therapy.	[35,39,40,109,110]
**TMB-H** **(Pembrolizumab)**	**NGS:** Validated, FDA-approved comprehensive genomic profiling to calculate mutations per megabase; may also determine MSI status simultaneously. **ctDNA:** Plasma-based liquid biopsy genomic profiling panels.	**Quantitative neoantigen selection:** Enrichment for patients with high predicted neoantigen burden. **Strict cutoff:** TMB ≥10 mut/Mb determined by validated genomic sequencing. **Multi-target analysis:** Broad-panel NGS captures both mutational burden and microsatellite status concurrently from a single sample.	Serves as a predictive immunotherapy marker in rare, highly mutated cancers such as neuroendocrine carcinomas. Expands trial options where standard cytotoxic chemotherapy has limited or refractory efficacy.	[42,110,111]
**HER2** **(Trastuzumab deruxtecan)**	**IHC:** Primary modality for surface expression and tissue stratification (high vs. low). **ISH:** Reflex verification for IHC 2+ cases. **NGS/ctDNA:** Tissue or liquid biopsy panels to evaluate multiple targets, somatic mutations, or amplifications concurrently.	**Expression-based stratification:** Cohort enrichment based on IHC 3+ or IHC 2+ status across multiple solid tumor cohorts. **ADC expansion:** Shift from binary to continuous, quantitative expression scoring to capture HER2-low and HER2-ultralow populations.	Broadens targeted options for rare, aggressive HER2-overexpressing histologies, including uterine serous carcinoma, salivary gland tumors, and biliary tract cancers. Maximizes the bystander effect of novel ADCs in low-expression cohorts.	[74,101,112]
**NTRK fusion** **(Entrectinib** **Larotrectinib** **Repotrectinib)**	**NGS:** Primary modality for multigene tissue panels; RNA-based NGS is generally preferred over DNA-based methods for detecting structural rearrangements. **FISH:** Rapid-turnaround alternative but lacks information on fusion structure. **DNA-based PCR:** Alternative when NGS is unavailable; detects known single-nucleotide alterations but misses low-variant-allele-frequency events.	Patients must harbor a documented, in-frame fusion involving NTRK1, NTRK2, or NTRK3. Because DNA-based NGS panels frequently miss large, complex intronic NTRK3 deletions, confirmatory RNA-based NGS or FISH is required for study inclusion.	Though rare across cancers overall, NTRK fusions are definitive drivers in ultra-rare tumors such as infantile fibrosarcoma and MASC.	[61,62,65,66]
**RET fusion** **(Selpercatinib)**	**DNA-based NGS:** High sensitivity; detects somatic mutations at low variant allele frequencies. RNA-based NGS: Confirmatory test, considered the gold standard. **FISH:** Rapid-turnaround alternative but lacks information on fusion structure. **DNA-based PCR:** Alternative when NGS is unavailable; limited to known single-nucleotide alterations. **IHC:** Screening tool for protein overexpression; not commonly used due to poor standardization and dependence on fusion partners.	**Kinase fusion enrichment**: Screens for RET rearrangements with known partners (e.g., KIF5B) to ensure precise oncogenic matching. **Lineage-stratified enrichment**: For NSCLC and papillary thyroid cancer cohorts, eligibility requires confirmed RET structural fusions via DNA/RNA NGS; for medullary thyroid cancer (MTC) cohorts, enrichment instead targets activating point mutations (e.g., RET M918T) via targeted DNA sequencing.	Drives durable disease control in non-canonical rare populations, including pancreatic adenocarcinoma and salivary gland tumors. Replaces older, poorly tolerated multikinase inhibitors (MKIs) with more selective RET inhibitors, improving progression-free survival in low-incidence, driver-positive populations.	[64,113]
**BRAF V600E** **(Dabrafenib + trametinib)**	**IHC:** Rapid screening using mutation-specific antibodies (e.g., VE1); limited to the V600E substitution and requires tissue confirmation. **PCR:** High-sensitivity, rapid-turnaround test for known codon 600 variants; narrower scope than comprehensive profiling. **NGS:** Gold-standard approach capturing BRAF alterations alongside co-occurring genomic drivers, with longer turnaround time. **ctDNA:** Non-invasive alternative useful when tissue is exhausted or inaccessible, though limited by tumor shedding rates and lower sensitivity for low-abundance alleles.	Mutation-specific enrichment: Targets the V600E substitution and rare non-V600 codon 600 variants (e.g., V600K, V600R) via DNA/RNA NGS or validated PCR/IHC. Safety gating: Confirms the valine-to-glutamic-acid substitution to prevent paradoxical MAPK activation during dual BRAF/MEK inhibition. Lineage-stratified basket design: Restricts eligibility to confirmed BRAF V600 alterations while accounting for differing baseline response rates across histology-specific rare cohorts (e.g., CNS vs. GI malignancies).	First combinational tumor-agnostic regimen approved for solid tumors; illustrates one approach to overcoming resistance via bypass mechanisms. Redefined treatment for anaplastic thyroid cancer (ATC); also active in biliary tract and low-grade serous ovarian cancers.	[52,107]

## 6. Regulatory and Collaborative Frameworks

Regulatory incentives and multinational collaborative frameworks have emerged as critical enablers of rare cancer research, addressing longstanding barriers related to drug development, diagnostic accuracy, and clinical trial feasibility. Legislative policies such as the U.S. Orphan Drug Act and parallel European regulations provide market exclusivity, reduced regulatory fees, grant support, and protocol assistance, thereby lowering financial risk and encouraging pharmaceutical investment in rare cancer therapeutics. Launched in May 2019, Project Orbis facilitates concurrent international evaluation of oncology treatments, accelerating global access. Additionally, the FDA Rare Oncology Agility Framework (ROAF) supports flexible evidence generation and adaptive approaches in rare cancers, complementing these initiatives. Collectively, these frameworks have been instrumental in translating early-phase and biomarker-driven trials into approved therapies, particularly in settings where traditional commercial interest is limited [27].

Beyond regulatory incentives, international and national consortia play an essential role in enabling rare cancer trials by facilitating rapid patient accrual, standardized molecular diagnostics, and centralized expert review. Central pathology review is particularly impactful, as diagnostic discordance between local and expert review remains common in rare tumors. In the French CONCART/CONTICANET sarcoma study, expert review resulted in diagnostic modification in approximately 30% of cases, with up to 50% discordance identified at the local level, directly affecting treatment selection and clinical trial eligibility [114]. These findings underscore the necessity of centralized expertise to ensure accurate diagnosis and trial integrity.

In the United States, the National Cancer Institute (NCI) serves as a central driver of rare cancer research through funding, infrastructure development, and coordination of multicenter efforts. Landmark initiatives such as NCI-Molecular Analysis for Therapy Choice (MATCH) have advanced tumor-agnostic precision oncology, while dedicated programs—including the My Pediatric and Adult Rare Tumor Network (MyPART), NCI-Comprehensive Oncology Network Evaluating Rare CNS Tumors (CONNECT) for rare central nervous system tumors, the Childhood Cancer Data Initiative (CCDI), and the Rare Cancer Data Registry—support collaborative research, data harmonization, and trial readiness [115]. MyPART, in particular, exemplifies a patient-centered, histology-agnostic model, demonstrating the feasibility of large-scale longitudinal data and biospecimen collection across multiple rare tumor types, with high data completeness. This framework supports the development of external control cohorts for single-arm trials, a critical strategy in ultra-rare cancers where randomized studies are not feasible [115,116].

European efforts similarly emphasize cross-border collaboration through structured expert networks. The European Reference Network for rare adult solid cancers (EURACAN) connects specialized centers across Europe to improve diagnostic accuracy, care standardization, and access to expertise, while leveraging epidemiologic and outcome data from the RARECARE database to monitor survival trends and treatment patterns. Complementing these efforts, the International Rare Cancers Initiative (IRCI) aligns priorities across major research organizations to enable multinational interventional trials in rare cancers that would be underpowered at the national level. By reducing duplication, accelerating trial activation, and improving methodological rigor, IRCI has significantly strengthened the global evidence base for rare cancer therapies [117].

International collaboration is often essential for adequately powered studies in ultra-rare cancers. To overcome the inherent challenges of patient accrual in ultra-rare cancers, a shift toward international collaboration and the integration of RWD is essential. In settings where traditional, large-scale trial designs are often infeasible, global networks facilitate adequately powered studies, while RWD provides critical external controls and natural history data to support regulatory decision-making and accelerate the delivery of precision therapies [118]. External or synthetic control arms derived from real-world datasets can provide meaningful comparators for single-arm studies. For example, a real-world external control arm was used in the single-arm LIBRETTO-001 trial evaluating selpercatinib in patients with RET-mutant medullary thyroid cancer [119]. These findings illustrate that appropriately weighted real-world control arms may be considered as a useful complement to conventional comparators in rare cancer trials.

**Figure 3:**
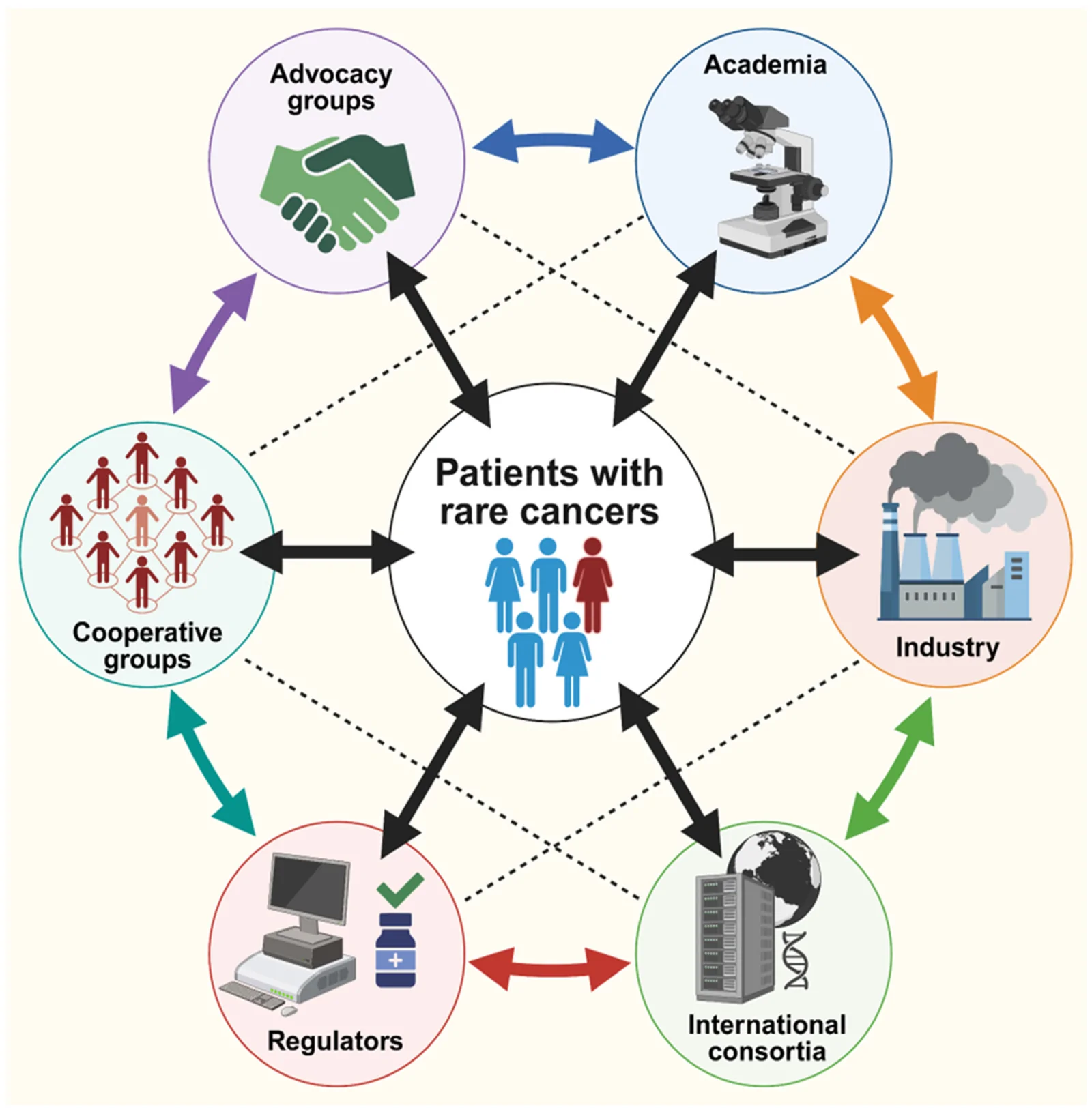
Collaborative Ecosystem of Rare Cancer Drug Development. Created in BioRender. Myat, Y. (2026) https://BioRender.com/2xyzuer.

Regulatory policies and collaborative networks collectively enable accurate diagnosis, multicenter trial feasibility, and rapid translation of findings into clinical care. Coordinated initiatives at the national and international level, alongside patient-centered networks, provide the infrastructure and methodological rigor essential for accelerating rare cancer research, supporting precision medicine approaches, and improving outcomes for patients with these understudied malignancies. The collaborative ecosystem of multiple agencies and clinicians is vital for rare cancer development (Figure 3). A multidisciplinary approach, including the involvement of physicians, patient groups, and industry may especially be beneficial in rare cancer groups.

Federal agencies outside traditional health research structures have also contributed meaningfully to this ecosystem. The U.S. Department of Defense (DOD), through the Congressionally Directed Medical Research Programs Rare Cancers Research Program (RCRP), supports innovative basic and translational research focused on disease biology, rare tumor–specific model development, therapeutic discovery, and shared research infrastructure [120]. By investing in centralized data platforms, biospecimen repositories, patient registries, and natural history studies, the RCRP enhances trial readiness and enables decentralized and digitally supported research approaches increasingly necessary for rare cancer populations [121].

This conceptual and simplified figure illustrates an overview of the interconnected ecosystem in rare cancer drug development. The collaboration between academia, industry, international consortia, regulators, cooperative groups, and advocacy groups are vital in helping patients with rare cancers, and especially for ultra-rare tumor indications. This conceptual overview omits specific institutional funding mechanisms, proprietary intellectual property agreements, and individual legal frameworks governing cross-sector partnerships. Created in BioRender. Myat, Y. (2026) https://BioRender.com/2xyzuer.

## 7. Future Directions and Roadmap for Accelerating Rare Cancer Drug Development

Accelerating drug development in rare cancers requires a coordinated roadmap that integrates advances in molecular diagnostics, innovative trial design, regulatory support, and global collaboration. To accelerate progress over the next decade, key stakeholders must address several intersecting clinical, regulatory, and economic challenges. Table 3 outlines the core actionable priorities and strategic directions for future rare cancer drug development. Addressing these priorities is critical to overcoming limited patient numbers, biological heterogeneity, and unmet clinical need.

### 7.1. Diagnostics and Molecular Characterization

Accelerating rare cancer drug development will require continued expansion of comprehensive molecular diagnostics and improved data-sharing across institutions. Broader implementation of next-generation sequencing (NGS), multigene expression assays, and companion diagnostics—particularly within pan-omic frameworks—will enable deeper characterization of rare tumors and identification of actionable alterations [124]. Concurrently, the future optimization of liquid biopsy approaches lies in moving beyond basic sequencing toward dynamic, longitudinal molecular monitoring of tumor evolution (Table 3). Utilizing serial ctDNA and multi-omic profiling will allow clinicians to track real-time clonal evolution, identify emerging resistance mechanisms before radiographic progression, and map temporal tumor heterogeneity, thereby supporting more adaptive and personalized treatment strategies [125].

Emerging technologies such as spatial transcriptomics, organoid platforms, and computational protein/drug design may serve as future enabling tools in precision oncology (Table 3). Spatial transcriptomics maps gene expression architecture, providing vital architectural clarity regarding the uncharacterized rare tumor microenvironment. For instance, Serra and authors were able to use spatial transcriptomics to identify four major tumor microenvironment subtypes of invasive lobular carcinoma [126]. Organoid platforms also serve as valuable preclinical models for studying tumor heterogeneity, clonal evolution, and tumor microenvironment interactions in human cancers. These systems are particularly useful when clinical tissue samples are limited, and several studies have demonstrated concordance between organoid-based drug responses and patient outcomes [127].

Finally, advanced computational protein and de novo drug design leverages machine-learning algorithms to predict and synthesize bespoke therapeutic molecules, accelerating drug discovery pipelines for novel, previously undruggable rare oncogenic drivers [128]. Moving beyond current retrospective data classification, the future of A.I in precision oncology lies in its capacity to act as a predictive, multi-dimensional modeling engine. By integrating genomic, transcriptomic, and clinical data layers, future A.I-driven frameworks may improve the detection of rare molecular alterations. This computational approach can help identify potential therapeutic targets while addressing the biological complexity and heterogeneity characteristic of rare tumor subtypes. While these frameworks offer potential to uncover actionable targets, their utility depends on validation across diverse rare tumor cohorts [103,104,129].

Although spatial profiling, organoids, and A.I may clarify rare-tumor biology, most applications remain preclinical or retrospective. Their clinical utility requires independent, prospective validation against standard pathology and sequencing; an organoid response should not be presented as a validated predictor of patient benefit [130]. Programs should also measure access to molecular testing, assay failure and turnaround time, and the proportion of screened patients who can enroll. Local laboratory visits, telehealth follow-up, and patient-advocacy input may reduce travel burdens where protocol safety and data quality permit [131,132].

While current tumor-agnostic therapeutic development has largely focused on solid tumors, similar oncogenic drivers are increasingly recognized in hematologic malignancies, highlighting an opportunity to bridge therapeutic strategies across tumor types [133]. Bridging these solid and hematologic therapeutic paradigms will pave the way for a new era of multi-omic, tumor-agnostic clinical trials.

### 7.2. Therapeutic Resistance and Disease Evolution

Tumor-agnostic approvals traditionally assume that a shared molecular alteration predicts uniform therapeutic benefit regardless of histology, yet efficacy often varies based on histology. This may be explained by lineage-specific factors, intratumor heterogeneity, and resistance mechanisms [122].

Resistance to targeted therapies remains a major challenge in rare cancers, particularly when oncogenic pathways are shared across multiple signaling networks. For example, combined BRAF and MEK inhibition with dabrafenib and trametinib was developed to address pathway reactivation within the MAPK cascade; however, resistance continues to emerge through alternative and adaptive mechanisms. Resistance to targeted therapies can arise through multiple mechanisms, including bypass signaling, secondary resistance mutations, lineage plasticity, and adaptation of the tumor microenvironment. These mechanisms reflect both on-target and off-target processes and can be broadly categorized into intrinsic and adaptive resistance. Even when a driver oncogene is effectively inhibited with monotherapy, tumors may continue to proliferate through activation of alternative oncogenic pathways or bypass signaling, involving molecular alterations that occur upstream, downstream, or directly at the level of the targeted protein. Secondary resistance mutations can emerge under therapeutic pressure or through clonal evolution, further diminishing drug efficacy over time [134].

This may explain why, despite agnostic approval, some cancer types with the same biomarker respond differently to the same treatment. Different clones and subclones of a tumor may acquire new mutations that emerge during treatment [122]. For instance, rapid EGFR-mediated feedback reactivation of the MAPK pathway is uniquely pronounced in colonic epithelium [135]. This resistance mechanism may explain why dabrafenib plus trametinib gained tumor-agnostic approvals for solid tumors except colorectal cancer (Table 1b). Furthermore, the high degree of heterogeneity in a small population of ultra-rare cancers makes it difficult to establish robust statistical significance without using a pooled population [20]. Consequently, ORR-based approvals may be limited by variable efficacy across different histologies.

In some cases, tumors undergo lineage plasticity in which the cancer shifts to an alternative cellular identity, often driven by epigenetic or transcriptional reprogramming; epithelial-to-mesenchymal transition is one such example observed in certain carcinomas. In some instances, lineage plasticity has been regarded as a new cancer hallmark [136]. Additionally, changes within the tumor microenvironment, including stromal and immune components, can modulate treatment response and contribute to therapeutic resistance [100].

Serial ctDNA analysis and repeat molecular profiling also play an important role in monitoring treatment response and identifying emerging resistance mechanisms over time (Table 3). For example, liquid biopsy studies evaluating resistance to BRAF inhibition in BRAF V600E-mutant non-small cell lung cancer (NSCLC) have demonstrated extensive genomic heterogeneity and dynamic clonal evolution during treatment [137].

These challenges are amplified in rare cancers, where disease biology and resistance pathways are less well characterized. As resistance evolves, tumors may further fragment into increasingly rare molecular subcohorts, compounding the scarcity of effective treatment options and underscoring the need for longitudinal molecular monitoring and adaptive therapeutic strategies.

### 7.3. Regulatory Strategy and Global Collaboration

Regulatory innovation and international collaboration will be essential to sustain progress in rare cancer therapeutics (Table 3). Expanded regulatory support, increased use of innovative trial designs, and broader acceptance of real-world evidence are critical to overcoming limitations posed by small patient populations. For ultra-rare indications, small single-arm phase II trials—such as those evaluating immunotherapy in advanced anal cancer—are often enough to demonstrate clinical utility and drive regulatory traction [138]. Moreover, when traditional histology-specific approvals are missing, established guideline and compendium updates provide vital mechanisms to secure off-label access for patients. Global initiatives such as NCI-CONNECT highlight both the promise and the challenges of rare cancer research, as access for participants outside the United States remains limited. Strengthening integration with international registries and cross-border data-sharing efforts will be necessary to enable adequately powered analyses and accelerate drug development [139]. This will be integral for equitable access to genomic testing and precision therapies across different healthcare systems and regions. Proposed solutions include universal genomic testing for all cancer patients, increased awareness to ensure timely identification of actionable alterations, expanded use of innovative and adaptive trial designs, and harmonized regulatory frameworks that facilitate global integration (Table 3).

Decentralized and digital clinical trial models will also serve as additional tools to improve global access and patient accrual in rare cancers. Decentralized and digitally enabled trial approaches allow for data collection in nontraditional settings. They represent important strategies to further improve access, recruitment and retention in rare cancer research (Table 3). By leveraging telemedicine, remote monitoring, electronic consent, and partnerships with local healthcare providers, these models reduce reliance on centralized trial sites and minimize patient travel burden. Several regulatory strategies are underway, including FDA’s Digital Health Center of Excellence as well as the European Medicines Agency (EMA) Network Strategy in 2025 further promote digital integration [140]. The growing role of decentralized/digital trial models aid in improving equitable access for geographically dispersed patients. Hybrid and fully decentralized designs also allow for more flexible and scalable study conduct, complementing innovative trial designs and supporting broader inclusion of underrepresented populations in clinical research.

Patient advocacy further facilitates early-phase clinical trial development and enrollment, particularly in rare cancers. Advocacy groups play a key role in building patient registries, disseminating trial information, and directing patients to enrolling centers, thereby helping to bridge the gap between the research community and patients. These efforts are especially important given the small and geographically dispersed populations characteristic of rare cancers [121,141]. In addition to improving recruitment, advocacy groups enhance trial retention and design by emphasizing patient experience, incorporating patient-reported outcomes, and ensuring the development of clinically meaningful, patient-centered endpoints [142]. They also help identify real-world barriers, including inequities in access to care, enabling more collaborative and patient-centered trial design and implementation.

Given the heterogeneity of rare cancer populations and the limited generalizability of clinical trial data, robust real-world evidence will play an increasingly important role in informing regulatory decision-making and optimizing patient care [139]. Beyond regulatory hurdles, rare cancer drug development encounters substantial health economic challenges, including out-of-pocket expenses and complex payer reimbursement barriers. Because targeted precision therapies and tumor-agnostic agents are developed within small, fragmented patient cohorts, manufacturers may frequently establish high acquisition and launch prices to offset developmental investments, which may present a heavy economic burden on patients, families, and healthcare institutions [143]. Furthermore, the reliance on single-arm trial designs and surrogate endpoints creates significant uncertainty for health technology assessments, often resulting in restrictive coverage policies, prior authorization hurdles, and profound regional disparities in patient access. Strategies may include implementation of novel value-based pricing structures, risk-sharing agreements, and coordinated international reimbursement frameworks to ensure that emerging innovations in rare oncology remain both sustainable and broadly accessible (Table 3) [123]. Together, these strategies outline a pragmatic roadmap to accelerate rare cancer drug development by improving diagnosis, overcoming resistance, and enabling broader access to precision therapies.

## 8. Conclusions

The diagnosis and treatment of rare cancers have been improving throughout the past decade. Early-phase oncology trials have transformed treatment for rare cancers, leveraging novel agents such as targeted therapies, immunotherapies, and antibody-drug conjugates. These innovations have enabled patients with diverse tumor types to benefit from precision medicine, moving beyond organ-specific treatment. Innovative trial designs—including basket, umbrella, and adaptive trials—have accelerated tumor-agnostic drug development by efficiently studying rare mutations across multiple histologies and supporting individualized therapy, even in small patient populations. These designs have allowed for integration of biomarkers in the diagnosis, evaluation, and treatment of rare cancers.

Challenges remain, including limited patient accrual, restricted trial access, treatment resistance, biomarker integration, and disparities in molecular testing infrastructure. Future developments in rare cancer research will likely involve the integration of precision oncology with global collaborative trial networks to improve biomarker discovery, refine patient stratification, and accelerate development of personalized therapies across molecularly defined tumor populations. As oncology increasingly adopts biomarker-driven and tumor-agnostic approaches, rare cancers may serve as an important model for broader precision oncology innovation. Collectively, these advances hold promise for expanding effective, individualized treatment options and improving outcomes for patients with rare cancers who continue to face substantial unmet clinical needs.

## Figures and Tables

**Table 1:** (**a**). Cross-Tumor Biomarker-Driven Trials for Immunotherapy Relevant to Rare Cancers. (**b**). Cross-Tumor Biomarker-Driven Trials for Targeted Therapies Relevant to Rare Cancers. (**c**). Cross-Tumor Biomarker-Driven Trials for Fusion Inhibitors Relevant to Rare Cancers.

(**a**)					
**Drug** **(Therapeutic Class)**	**Clinical Trial Name (Phase)** **[ClinicalTrials Number]**	**Cancer Types Included**	**Key Efficacy Outcomes**	**Regulatory Status**	**Ref.**
Pembrolizumab (PD-1 inhibitor) targeting dMMR/MSI-H	KEYNOTE-051 (phase I/II) [NCT02332668] KEYNOTE-158 (phase II) [NCT02628067] KEYNOTE-164 (phase II) [NCT02460198]	CRC, non-CRC	ORR: 33.3% PR: 23% CR: 10.3% mDOR: 63.2 months	FDA granted accelerated approval for MSI-H/dMMR solid tumors on 23 May 2017 and full approval on 29 March 2023	[34]
	KEYNOTE- 158 (phase II) [NCT02628067]	Endometrial cancer, gastric cancer, small intestine cancer, ovarian cancer, cholangiocarcinoma, pancreatic cancer, brain cancer, sarcoma, breast cancer, other (pooled) cancer, cervical cancer, neuroendocrine cancer, prostate cancer, adrenocortical cancer, mesothelioma, thyroid cancer, SCLC, urothelial cancer, salivary cancer, renal cancer.	ORR: 33.8% PR: 23.1% CR: 10.7% mDOR: 63.2 months mPFS: 4 months mOS: 19.8 months		[35]
	KEYNOTE-016 (phase II) [NCT01876511]	Cohort A—CRC Cohort C—Non-CRC, including: endometrial cancer, pancreatic cancer, small intestinal cancer, gastroesophageal cancer, biliary cancer, ampullary cancer, and other cancer subtypes.	Cohort A - ORR: 56.1%, PR: 27%, CR: 29%, mPFS: 38.8 months, mOS: 80.8 months Cohort C - ORR: 59.6%, PR: 25%, CR: 34%, mPFS: 20.5 months, mOS: 86.4 months		[36]
Dostarlimab (PD-1 inhibitor) targeting dMMR	GARNET (phase I) [NCT02715284]	CRC Non-CRC, including: Small intestinal cancer, gastric cancer, pancreatic cancer, liver cancer, ovarian cancer, adrenal cortical cancer, biliary neoplasm, breast cancer, esophageal cancer, gallbladder cancer, genital neoplasm, pleural cancer, renal cell carcinoma, unknown origin cancer.	Overall ORR: 38.7% ORR for colorectal cancer: 36.2% ORR for non-colorectal cancer: 43.1%	FDA granted accelerated approval for dMMR solid tumors on 17 August 2021	[37]
Dostarlimab (PD-1 inhibitor) targeting dMMR	GARNET (phase I) [NCT02715284]	Endometrial cancer Non-endometrial cancers including: CRC, gastric cancer, small intestinal cancer, pancreatic carcinoma, ovarian cancer, and other cancer subtypes.	ORR: 44.1%, PR: 30.8%, CR: 13.3%, mPFS: 7 months		[38]
Pembrolizumab (PD-1 inhibitor) targeting TMB-H (≥10 mut/Mb)	KEYNOTE-158 (phase II) [NCT02628067]	Anal cancer, biliary cancer, cervical cancer, endometrial cancer, mesothelioma, neuroendocrine cancer, salivary cancer, SCLC, thyroid cancer, vulvar cancer.	ORR: 28%, PR: 25%, CR: 4%	FDA granted accelerated approval for TMB-H (≥10 mutations/megabase) solid tumors on 16 June 2020	[39]
Nivolumab and ipilimumab (Dual Anti-CTLA-4 and Anti-PD-1 inhibitors) targeting CTLA-4 and PD-1	DART Trial (SWOG1609) (phase II) [NCT02834013]	≥40 rare epithelial, neuroendocrine, and mesenchymal tumor types spanning head/neck, GI, GU, and gynecologic sites—see for full list.	ORR: 12%, mPFS: 2.1 months	No tumor-agnostic FDA approvals	[40]
(**b**)					
**Drug** **(Therapeutic Class)**	**Clinical Trial Name (Phase)** **[ClinicalTrials Number]**	**Cancer Types Included**	**Key Efficacy Outcomes**	**Regulatory Status**	**Ref.**
Dabrafenib + Trametinib (BRAF/MEK inhibitor) targeting BRAF V600E	NCI-MATCH (phase II) [NCT02465060] ROAR (phase II) [NCT02034110]	Biliary tract cancer; high-grade glioma; low-grade glioma; low-grade serous ovarian carcinoma; small intestinal adenocarcinoma; pancreatic adenocarcinoma; adenoneuroendocrine carcinoma; neuroendocrine carcinoma of the colon; mandibular ameloblastoma; combined small cell squamous carcinoma of the lung; peritoneal serous adenocarcinoma (mucinous papillary subtype); anal cancer; GIST.	ORR: 41%	FDA granted approval for BRAF V600E solid tumors in June 2022	[16,17,41]
Trastuzumab deruxtecan (ADC) targeting HER2	DESTINY-PanTumor02 (phase II) [NCT04482309] DESTINY-Lung01 (phase II) [NCT03505710] DESTINY-CRC02 (phase II) [NCT04744831]	Endometrial cancer, cervical cancer, ovarian cancer, bladder cancer, biliary tract cancer, pancreatic cancer, and other cancers (including adenocarcinoid tumor of the appendix, adenoid cystic carcinoma, salivary gland cancer, extramammary Paget disease, head and neck cancer, lip and/or oral cavity cancer, oropharyngeal neoplasm, intestinal adenocarcinoma, malignant neoplasm of unknown primary site, cutaneous melanoma, esophageal adenocarcinoma, esophageal squamous cell carcinoma, testis cancer, and vulva cancer).	DESTINY-PanTumor02—ORR: 51.4%, mDOR: 19.4 months DESTINY-Lung01—ORR: 52.9%, mDOR: 6.9 months DESTINY-CRC02—ORR: 46.9%, mDOR 5.5 months	FDA granted accelerated approval for HER2-positive (IHC 3+) solid tumors on April 5, 2024	[42,43]
Sotorasib (KRAS G12C inhibitor) targeting KRAS G12C	CodeBreak100 (phase I/II) [NCT03600883]	NSCLC, CRC, other cancer types (including pancreatic cancer, endometrial cancer, appendiceal cancer, and melanoma).	NSCLC—ORR: 32.2%, PR: 32.2%, CR: 0% CRC—ORR: 7.1%, PR: 7.1%, CR: 0% Other—ORR: 14.3%, PR: 14.3%, CR: 0%	No tumor-agnostic FDA approvals	[44]
Adagrasib (KRAS G12C inhibitor) targeting KRAS G12C	KRYSTAL-1 (phase I) [NCT03785249]	Pancreatic ductal adenocarcinoma, biliary tract cancer, cholangiocarcinoma, ampullary cancer, gallbladder cancer, other cancers of gastrointestinal origin, appendiceal cancer, small bowel cancer, gastroesophageal junction/esophageal cancer, gynecologic cancer, ovarian cancer, endometrial cancer, other cancer, unknown primary cancer, breast cancer.	ORR: 35.1%, PR: 35.1%, CR: 0%, mDOR: 5.3 months, mPFS: 7.4 months, mOS: 14 months	No tumor-agnostic FDA approvals	[45]
Erdafitinib (FGFR inhibitor) targeting FGFR 1-4 alterations	RAGNAR (phase II) [NCT04083976]	Cholangiocarcinoma, high-grade glioma, pancreatic cancer, breast cancer, squamous-cell head and neck cancer, NSCLC, carcinoma of unknown primary, CRC, endometrial cancer, esophageal cancer, gastric cancer, ovarian cancer, low-grade glioma, cervical cancer, salivary gland cancer, soft-tissue sarcoma, prostate cancer, other cancers.	ORR: 30%, PR: 27%, CR: 3%, mDOR: 6.9 months, mPFS: 4.2 months, mOS: 10.7 months	No tumor-agnostic FDA approvals	[46]
Zenocutuzumab (HER2/HER3 bispecific antibody) targeting NRG1 fusion	eNRG1 (phase II) [NCT02912949]	NSCLC, pancreatic ductal adenocarcinoma, cholangiocarcinoma, breast cancer, CRC, Cancer of unknown primary, endometrial sarcoma cancer, gastric cancer, ovarian cancer, renal cell carcinoma.	ORR: 30% mDOR: 11.1 months mPFS: 6.8 months	No tumor-agnostic FDA approvals	[47,48]
(**c**)					
**Drug** **(Therapeutic Class)**	**Clinical Trial Name (Phase)** **[ClinicalTrials Number]**	**Cancer Types Included**	**Key Efficacy Outcomes**	**Regulatory Status**	**Ref.**
Larotrectinib (NTRK fusion inhibitor) targeting NTRK fusion	LOXO-TRK-14001 (phase I/II) [NCT02122913] NAVIGATE (phase II) [NCT02576431] SCOUT (phase I/II) [NCT02637687]	Infantile fibrosarcoma; GIST; soft tissue sarcomas other than infantile fibrosarcoma or GIST; thyroid cancer; salivary gland cancer; lung cancer; CRC; melanoma; breast cancer; bone sarcoma; cholangiocarcinoma; pancreatic cancer; appendiceal cancer; congenital mesoblastic nephroma; hepatocellular cancer; and cancer of unknown primary.	ORR: 71% mDOR: 35.2 months mPFS: 28.3 months mOS: 44.4 months	FDA granted approval for NTRK fusion-positive solid tumors in November 2018	[49]
Entrectinib (NTRK fusion inhibitor) targeting NTRK fusion (other targets: ROS1, ALK)	ALKA-372-00 (phase I) [2012-000148-88] * STARTRK-1 (phase I) [NCT02097810] STARTRK-2 (phase II) [NCT02568267]	Sarcoma; mammary analog secretory carcinoma; NSCLC; thyroid cancer; colorectal cancer; breast cancer; neuroendocrine tumor; pancreatic cancer; cancer of unknown primary; gynecologic cancer; head and neck cancer; cholangiocarcinoma; upper gastrointestinal adenocarcinoma; and neuroblastoma.	ORR: 61.5% mDOR: 20 months mPFS: 13.8 months mOS: 33.8 months	FDA granted approval for NTRK fusion-positive solid tumors in August 2019	[50]
Selpercatinib (RET fusion inhibitor) targeting RET fusion	LIBRETTO-001 (phase I/II) [NCT03157128]	Pancreatic cancer; CRC; salivary gland cancer; cancer of unknown primary; breast cancer; sarcoma; xanthogranuloma; carcinoid; ovarian cancer; small intestinal cancer; cholangiocarcinoma; pulmonary carcinosarcoma; rectal neuroendocrine tumor; and carcinoma of the skin.	ORR: 43.9% mDOR: 24.5 months mPFS: 13.2 months mOS: 18 months	FDA granted approval for RET fusion-positive solid tumors in September 2022	[51]
Repotrectinib (ROS1 and TRK inhibitor) targeting NTRK fusion (other target: ROS1)	TRIDENT-1 (phase I/II) [NCT03093116]	NSCLC, salivary gland cancer, secretory carcinoma, soft tissue sarcoma, thyroid cancer, glioblastoma, cholangiocarcinoma, CRC, peripheral nerve sheath tumor, neuroendocrine tumor, pancreatic cancer, breast cancer, and other cancers (including gallbladder cancer and unknown primary cancer).	TRK-pretreated—ORR: 50%, mDOR: 9.9 months TKI-naive—ORR: 58%, mDOR: NE	FDA granted accelerated approval for NTRK fusion-positive solid tumors on 13 June 2024	[52,53]

* ALKA-372-00 is not registered on ClinicalTrials.gov and does not have a standard NCT number. It is a European clinical trial registered under the EU Clinical Trials Register with EudraCT number 2012-000148-88. **Abbreviations:** ADC, Antibody-drug conjugate; BRAF, v-Raf murine sarcoma viral oncogene homolog B1; MEK, mitogen-activated protein kinase kinase; BRAF V600E, valine-to-glutamic acid substitution at codon 600 in BRAF; NCI-MATCH, National Cancer Institute Molecular Analysis for Therapy Choice; ROAR, Rare Oncology Agnostic Research; NTRK, neurotrophic tropomyosin receptor kinase; ROS1, ROS proto-oncogene 1; ALK, anaplastic lymphoma kinase; RET, rearranged during transfection; HER2, human epidermal growth factor receptor 2; HER3, human epidermal growth factor receptor 3; FGFR, fibroblast growth factor receptor; KRAS, Kirsten rat sarcoma viral oncogene homolog; G12C, glycine-to-cysteine substitution at codon 12; PD-1, programmed cell death protein 1; CTLA-4, cytotoxic T-lymphocyte–associated antigen 4; dMMR, deficient mismatch repair; MSI-H, microsatellite instability–high; TMB-H, tumor mutational burden–high; NRG1, neuregulin 1; GIST, gastrointestinal stromal tumor; NSCLC, non–small cell lung cancer; CRC, colorectal cancer; ORR, objective response rate; CR, complete response; PR, partial response; mDOR, median duration of response; mPFS, median progression-free survival; mOS, median overall survival; TKI, tyrosine kinase inhibitor; FDA, U.S. Food and Drug Administration; DART, Dual Anti-CTLA-4 & Anti-PD-1 blockade in Rare Tumors; and eNRG1, NRG1 fusion-enriched cohort study; SCLC, small-cell lung cancer.

**Table 3:** Priorities for future directions in rare cancer drug development.

Domain	Key Priorities/Challenges	Strategic Direction and Future Solutions	Ref.
Clinical trial design factors	Small patient cohorts Difficulty enrolling patients	Expand multi-institutional basket/umbrella protocols and decentralized/digital trial models (telemedicine, remote monitoring, local provider partnerships) to improve patient access and recruitment	[12,15,25]
Regulatory factors	Single-arm data limitations Surrogate endpoints Geographic disparities	Leverage real-world evidence, guideline and compendium updates for off-label access, patient advocacy groups and harmonized global frameworks, such as integrating international registries	[13,27,118]
Diagnostic and molecular factors	Heterogenous efficacy Resistance mechanisms Limited tissue availability	Increase awareness of variable efficacy across different histologies despite tumor-agnostic treatments Consider integration of pan-omic frameworks, spatial transcriptomics, organoid platforms, and longitudinal liquid biopsies (serial ctDNA) to map real-time clonal evolution and resistance	[104,122]
Health economic factors	High acquisition/launch prices Payer coverage restrictions Testing equity low-variant-allele-frequency events.	Implement novel value-based pricing structures, risk-sharing agreements, and coordinated international health technology assessments to safeguard affordability and broad patient access	[8,123]

## Data Availability

No new data were created or analyzed in this study. Data sharing is not applicable to this article.

## Abbreviations

The following abbreviations are used in this manuscript:

ACT	Adoptive Cellular Therapy
ADC	Antibody-Drug Conjugate
ALK	Anaplastic Lymphoma Kinase
A.I	Artificial Intelligence
B7-H3	B7 homolog 3 protein
BRAF	v-raf murine sarcoma viral oncogene homolog B1
BsAb	Bispecific Antibody
CAR-T	Chimeric Antigen Receptor T-cell therapy
CAR-NK	Chimeric Antigen Receptor Natural Killer cell therapy
CCDI	Childhood Cancer Data Initiative
CNS	Central Nervous System
CONNECT	Comprehensive Oncology Network Evaluating Rare CNS Tumors
CRC	Colorectal Cancer
CTLA-4	Cytotoxic T-lymphocyte antigen 4
ctDNA	Circulating Tumor DNA
DART	Dual Anti-CTLA-4 & Anti-PD-1 blockade in Rare Tumors
dMMR	Deficient Mismatch Repair
DOD	U.S. Department of Defense
EGFR	Epidermal Growth Factor Receptor
EMA	European Medicines Agency
EURACAN	European Reference Network for rare adult solid cancers
FDA	U.S. Food and Drug Administration
FGFR	Fibroblast Growth Factor Receptor
FISH	Fluorescence in Situ Hybridization
HER2	Human Epidermal Growth Factor Receptor 2
HIF-2α	Hypoxia-Inducible Factor 2 alpha
IARC	International Agency for Research on Cancer
ICD-O	International Classification of Diseases for Oncology
IHC	Immunohistochemistry
IRCI	International Rare Cancers Initiative
MAPK	Mitogen-Activated Protein Kinase
MATCH	National Cancer Institute-Molecular Analysis for Therapy Choice
mDOR	Median duration of response
MEK	Mitogen-Activated Protein Kinase Kinase
mPFS	median progression-free survival
MSI-H	Microsatellite Instability–High
mOS	median overall survival
MTC	Medullary Thyroid Cancer
MyPART	My Pediatric and Adult Rare Tumor Network
NSCLC	non-small cell lung cancer
NGS	Next-Generation Sequencing
NK	Natural Killer
NCI	National Cancer Institute
NRG1	Neuregulin 1
NTRK	Neurotrophic Tyrosine Receptor Kinase
ORR	Objective Response Rate
PD-1	Programmed cell death protein 1
PD-L1	Programmed death-ligand 1
RCRP	Rare Cancers Research Program
RCT	Randomized Controlled Trial
RET	Rearranged during Transfection
ROAF	Rare Oncology Agility Framework
ROAR	Rare Oncology Agnostic Research
ROS1	ROS proto-oncogene 1
RWD	Real-world data
TCR	T-cell Receptor
TCR-T	T-cell Receptor engineered T-cell therapy
T-Dxd	Trastuzumab-Deruxtecan
T-DM1	Trastuzumab-Emtansine
TIL	Tumor-Infiltrating Lymphocytes
TKI	Tyrosine kinase inhibitor
TMB	Tumor Mutational Burden
TMB-H	Tumor Mutational Burden-High
TME	Tumor Microenvironment
WHO	World Health Organization
